# A gene expression signature of RAS pathway dependence predicts response to PI3K and RAS pathway inhibitors and expands the population of RAS pathway activated tumors

**DOI:** 10.1186/1755-8794-3-26

**Published:** 2010-06-30

**Authors:** Andrey Loboda, Michael Nebozhyn, Rich Klinghoffer, Jason Frazier, Michael Chastain, William Arthur, Brian Roberts, Theresa Zhang, Melissa Chenard, Brian Haines, Jannik Andersen, Kumiko Nagashima, Cloud Paweletz, Bethany Lynch, Igor Feldman, Hongyue Dai, Pearl Huang, James Watters

**Affiliations:** 1 Department of Molecular Profiling and Research Informatics, Merck Research Laboratories, West Point, Pennsylvania 19486, USA; 2 Rosetta Inpharmatics LLC, a wholly-owned subsidiary of Merck & Co., Inc, Seattle, Washington 98109, USA; 3 Oncology basic research, Merck Research Laboratories, 33 Avenue Louis Pasteur Boston, MA, USA 02115; 4 Oncology, Merck Research Laboratories, North Wales, Pennsylvania, 19454, USA

## Abstract

**Background:**

Hyperactivation of the Ras signaling pathway is a driver of many cancers, and RAS pathway activation can predict response to targeted therapies. Therefore, optimal methods for measuring Ras pathway activation are critical. The main focus of our work was to develop a gene expression signature that is predictive of RAS pathway dependence.

**Methods:**

We used the coherent expression of RAS pathway-related genes across multiple datasets to derive a RAS pathway gene expression signature and generate RAS pathway activation scores in pre-clinical cancer models and human tumors. We then related this signature to KRAS mutation status and drug response data in pre-clinical and clinical datasets.

**Results:**

The RAS signature score is predictive of KRAS mutation status in lung tumors and cell lines with high (> 90%) sensitivity but relatively low (50%) specificity due to samples that have apparent RAS pathway activation in the absence of a KRAS mutation. In lung and breast cancer cell line panels, the RAS pathway signature score correlates with pMEK and pERK expression, and predicts resistance to AKT inhibition and sensitivity to MEK inhibition within both KRAS mutant and KRAS wild-type groups. The RAS pathway signature is upregulated in breast cancer cell lines that have acquired resistance to AKT inhibition, and is downregulated by inhibition of MEK. In lung cancer cell lines knockdown of KRAS using siRNA demonstrates that the RAS pathway signature is a better measure of dependence on RAS compared to KRAS mutation status. In human tumors, the RAS pathway signature is elevated in ER negative breast tumors and lung adenocarcinomas, and predicts resistance to cetuximab in metastatic colorectal cancer.

**Conclusions:**

These data demonstrate that the RAS pathway signature is superior to KRAS mutation status for the prediction of dependence on RAS signaling, can predict response to PI3K and RAS pathway inhibitors, and is likely to have the most clinical utility in lung and breast tumors.

## Background

Signal transduction in response to growth factor receptor activation in tumors is a complex process that involves downstream signaling through the RAS (reviewed in [1]) and PI3K (reviewed in [2]) signaling pathways. These pathways are among the best characterized in cancer biology, involve a network of protein and lipid kinases working in concert to regulate diverse biological outputs, and can be activated by multiple mechanisms including gene amplification and somatic mutation. Understanding the role of these pathways in cancer biology has been enabled through the characterization of alterations in component pathway nodes including amplification of receptor tyrosine kinases like Her and EGFR, and genetic changes in PTEN, PIK3CA, AKT, KRas and BRAF, all of which have been shown to contribute to the cancer phenotype. The RAS and PI3K pathways are thought to work in parallel and/or through cross-talk such that optimal therapeutic benefit can be achieved only through inhibition of both pathways. As AKT is a central node in the PI3K pathway and MEK is a central node in the RAS pathway, developing inhibitors of AKT and MEK is a strategy being pursued by the pharmaceutical industry [3].

Recent clinical data have emerged demonstrating that activating mutations in the KRAS gene predict resistance to treatment with inhibitors of the epidermal growth factor receptor (EGFR). For example, KRAS mutations are associated with decreased disease control rate, shorter progression-free survival and reduced overall survival in patients with advanced or metastatic colorectal cancer treated with the EGFR-targeting antibodies cetuximab or panitumumab [4-6]. In non-small cell lung cancer, the relationship between KRAS mutation and response to EGFR inhibitors is less clear. Response rates in patients that do not harbor an activating mutation in EGFR are low, and mutations in KRAS and EGFR rarely occur in the same tumor. As such, there has been no clear relationship between KRAS mutation status and clinical outcomes in patients treated with the EGFR tyrosine kinase inhibitors gefitinib or erlotinib [7]. Therefore, while alterations in specific RAS pathway components have lead to an increased understanding of the molecular drivers of response to EGFR inhibition in colorectal cancer, the relationship between KRAS mutation, RAS pathway dependence, and drug response is less clear in NSCLC and other tumor types.

Given the importance of KRAS activation for the selection of targeted cancer therapies, it is crucial that optimal methods are developed to measure the activation state of RAS in tumors. Due to the numerous genetic changes in tumors and the complexity of mechanisms underlying RAS pathway activation, a more comprehensive means of assessing RAS pathway activation status would be preferable. One way to enable a more comprehensive readout of pathway activity is to identify gene expression profiles that are indicative of pathway activation status. A gene expression signature-based pathway readout may be more appropriate than relying on a single indicator of pathway activity, as alterations in multiple signaling components could lead to pathway activation and result in similar downstream effects (for example, mutations in B-raf also lead to pathway activation and may lead to resistance to therapies targeting EGFR in colorectal cancer [4,5,8]).

Recent gene expression profiling efforts have identified pathway signatures that can be applied broadly across different datasets to monitor pathway activity. Moreover, recent studies have shown that pathway signatures can predict drug response *in vitro *and stratify tumors according to predicted pathway status [9-13]. Gene expression signatures could have additional benefit as pathway biomarkers, as these signatures could be used for both pretreatment patient stratification (i.e. prospectively identifying patients harboring tumors that are dependent on RAS signaling) and pharmacodynamic evaluation (i.e. monitoring pathway inhibition post-treatment). However, comparing pathway signatures to one another and assessing their robustness in independent datasets can be hampered by the use of heterogeneous microarray profiling and analysis methods [14-16]. Given the clinical importance of understanding RAS pathway activation and its relationship to drug response, our main goal was to develop a gene expression signature indicative of RAS pathway activity in human tumors that is robust and translatable across multiple tumor types and datasets. With such a tool in hand, it would be possible to assign a RAS activation score to tumors for the purposes of drug response prediction and pharmacodynamic assessment.

Through an integrated analysis of literature data and internal datasets incorporating both cell line models and human tumors, we identified a RAS pathway signature consisting of 147 genes that is coherently expressed across multiple datasets. The RAS pathway signature has a high sensitivity for detecting KRAS mutant cell lines and human tumors, but also identifies samples that have apparent RAS pathway activation in the absence of a KRAS mutation. We show that baseline levels of the RAS pathway signature predict resistance to AKT inhibition and sensitivity to MEK inhibition in cell line panels independent of KRAS mutation status, that the signature is downregulated by MEK inhibition, and that the signature is a better predictor of RAS pathway dependence compared to KRAS mutation status in lung cancer cell lines. In human tumors, the RAS signature is coherent across multiple tumor types, is elevated in clinical subtypes not known to harbor KRAS mutations (i.e. ER negative breast cancer), and predicts resistance to cetuximab in metastatic colorectal cancer. These data demonstrate that the RAS signature significantly expands the population of human tumors exhibiting RAS pathway deregulation, and has potential clinical utility in identifying lung and breast tumors where RAS pathway dependence should be considered when choosing appropriate targeted therapies.

## Results and Discussion

### Development of a novel RAS pathway gene expression signature

We were interested in the possibility that quantification of Ras-dependent gene expression would provide a better measure of Ras activity in cancer cells than mutation analysis. To develop a gene expression signature of Ras activity, we started with published RAS pathway signatures generated using different model systems by three laboratories [9,12,17]. All of the signatures were split into two opposite "arms" - the "up" arm, which is upregulated, and the "down" arm, which is downregulated, as signaling through the RAS pathway increases. While these three signatures report on a similar biological state, the signatures contain different genes, and the expression of many of the genes within each signature did not adhere to the expected correlation pattern in several publicly available tumor profiling datasets (Additional file 1, Figure S1). Therefore, these signatures may not provide a robust measurement in clinical samples.

We then identified a new RAS pathway signature by assembling the genes from the up arms of these three signatures into a "superset" of 812 genes. By assessing the correlation of genes within this superset in publicly available lung, breast, and colon gene expression datasets, we identified a coherent subset of genes that were significantly correlated with each other in all datasets (see methods; Additional file 2, Figure S2). The genes that belonged to this subset across all the datasets were selected as the up arm of our RAS pathway signature. Genes significantly anticorrelated with the up arm across a lung cancer cell line panel were then selected as the down arm. This procedure resulted in the identification of a 147 gene signature (105 up genes, 42 down genes, Additional file 3, Table S1) that we call the "RAS pathway signature". At least 20 genes in this signature are established components of the the RAS-MEK-ERK signaling network. These include multiple transcription factors and targets of ERK signaling such as *FOS *and *IER3 *as well as MAPK-phosphatases and sprouty genes involved in feedback inhibition of MEK/ERK signaling (dual specificity phosphatases 6 (*DUSP6*), *DUSP1, DUSP4, DUSP5 *and *SPRY4*)) [18].

### RAS pathway signature coherence

We first assessed the coherence of our RAS pathway signature in publicly available colon, lung, and breast tumor datasets that were independent of those used to discover the signature (see materials and methods). The coherence test incorporates the knowledge that a signature is comprised of two oppositely regulated components (i.e. up and down arms) in order to assess the robustness of the signature when it is analyzed in independent datasets. The signature was significantly coherent across all tumor types, with each of these datasets having a p-value less than 10^-5 ^for the coherence test (Additional file 4, Table S2). We conclude that the RAS pathway signature is a robust signature that is translatable across tumor types, including tumors in which the prevalence of mutations in KRAS is low (i.e. breast cancer).

### RAS pathway signature score calculation and prediction of KRAS mutational status

We calculated a composite score for our RAS pathway signature as described in [19]. Briefly, we used the following unweighted averaging scheme to calculate signature scores: First, individual datasets were mean normalized and gene expression for each gene in each sample was expressed as the log(10) ratio relative to the mean. Signature scores were then determined by calculating the mean log(10) ratio of genes in the "up" branch minus the mean log(10) ratio of genes in the "down" branch.

**Figure 1:**
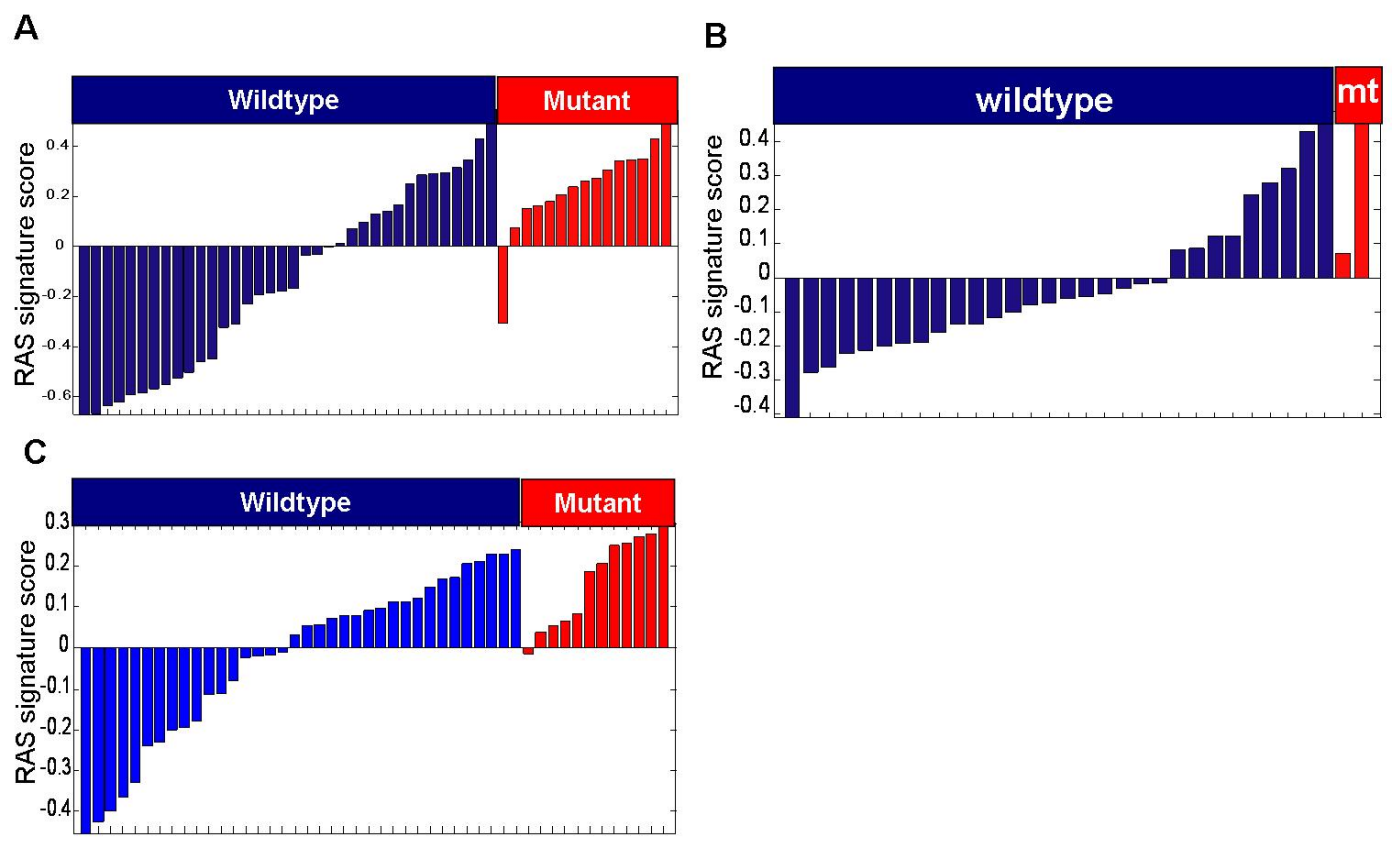
**RAS signature score relationship to Kras mutation status**. RAS signature scores relative to KRAS mutation status in (A) lung cancer cell lines, (B) breast cancer cell lines, and (C) lung tumors. The Y-axis shows the RAS pathway signature score relative to the mean of all samples in the experiment. Samples in red are KRAS mutant, samples in blue are KRAS wild-type

We then assessed the ability of the RAS pathway signature score to predict KRAS mutation status in lung cancer cell lines (n = 50), breast cancer cell lines (n = 32), and lung tumors (n = 48). Using a signature score of zero (mean value across a population) as the threshold, 14/15 lung cancer cell lines, 2/2 breast cancer cell lines, and 11/12 lung tumors with KRAS mutations had high signature scores (Figure 1). Interestingly, a significant number of KRAS wild-type cell lines and tumors exhibited high RAS pathway signature scores (40% of wild-type lung cell lines, 30% of wild-type breast cell lines, and 53% of wild-type lung tumors), suggesting that these samples have upregulated RAS signaling through another mechanism. Thus, in these datasets the RAS pathway signature is a high sensitivity but low specificity of predictor of KRAS mutation status, indicating that using the RAS pathway signature as a predictor of RAS pathway deregulation would significantly increase the population of "RAS pathway active" lung or breast tumors compared to using KRAS mutation status.

### Baseline level of RAS pathway signature correlates with drug sensitivity independent of KRAS mutation status

**Figure 2:**
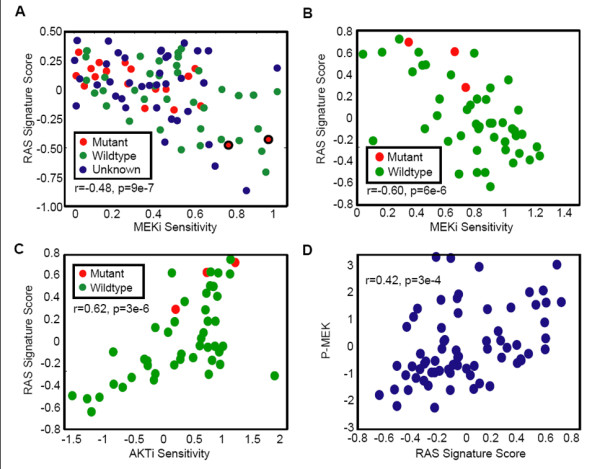
**RAS signature versus drug sensitivity or pathway activation**. RAS signature score (Y-axis) versus cell sensitivity to a MEK inhibition (X-axis) across a panel of (A) lung or (B) breast cell lines. Lower numbers on the X-axis indicate increasing sensitivity. Cell lines circled in bold in (A) have a KRAS mutation but have low signature scores and are resistant to MEK inhibition. (C) RAS signature score (Y-axis) versus cell sensitivity to an AKT inhibitor (X-axis) across a panel of breast cancer cell lines. Lower numbers on the X-axis indicate increasing sensitivity. (D) phospho-MEK (Y-axis) versus RAS signature score (X-axis) across a panel of breast cancer cell lines. In all cases, the RAS signature score was calculated relative to the mean of all cell lines in the respective experiment. R = Pearson correlation coefficient, p = the corresponding P-value.

We next assessed the relationship between baseline levels of the RAS pathway signature and cell line sensitivity to small molecule inhibitors of AKT and MEK in independent experiments involving panels of lung cancer (n = 93) and breast cancer (n = 69) cell lines. Across lung cancer cell lines, elevated RAS pathway signature score at baseline was significantly correlated with sensitivity to a small molecule inhibitor of MEK1 and MEK2 (PD325901; Figure 2A). Interestingly, this correlation was observed within both KRAS mutant and KRAS wild-type cell lines. In addition, two lung cancer cell lines with KRAS mutations exhibited low RAS pathway signature scores and were resistant to MEK inhibition, suggesting that while they are KRAS mutant, they are not dependent on RAS signaling. In breast cancer cell lines, only 3 of which are known to harbor a KRAS mutation, baseline levels of the RAS pathway signature were again significantly correlated with sensitivity to MEK inhibition, and were also significantly correlated with resistance to a small molecule inhibitor of AKT (MK-2206; Figure 2B, 2C).

In addition to gene expression profiling of these cell lines, we also assessed the activation state of key RAS pathway signaling nodes in both the lung and breast cancer cell line panels using reverse phase protein microarrays [20]. The RAS pathway signature score was significantly correlated with pMEK and pERK in both lung and breast cancer cell lines, with the highest correlation observed for pMEK in breast cancer lines (Figure 2D). Taken together, these results suggest that the RAS pathway signature integrates information transmitted through upstream activation of RAS signaling, correlates with phospho-protein readouts of RAS signaling, and is a better predictor of response to agents targeting MEK and AKT compared to KRAS mutation status in lung and breast cancer cell lines.

### Acquired resistance to AKT inhibition is associated with upregulated RAS signature

**Figure 3:**
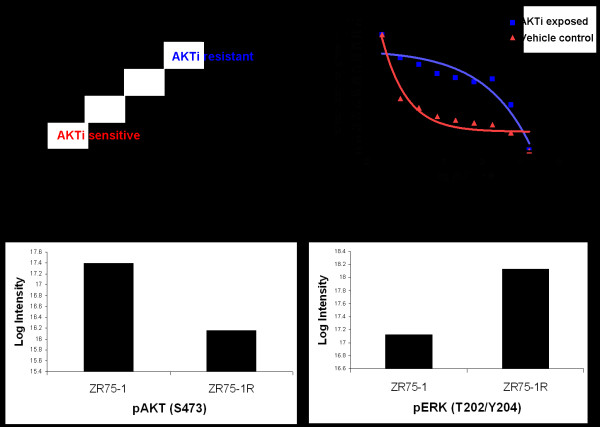
**Acquired resistance to AKT inhibition is associated with increased RAS signature**. (A) generation of a cell line with acquired resistance to MK-2206 by culturing in increasing drug concentrations over 7 months. (B) Cells cultured in vehicle for 7 months remain sensitive to AKT inhibition. Cells with acquired resistance to MK-2206 show decreased pAKT (C) and increased pERK (D).

To further explore the relationship between the RAS pathway signature and resistance to AKT inhibition, we generated a resistant version of an MK-2206 sensitive breast cancer cell line (ZR-75-1) by exposure to increasing concentrations of MK-2206 for a period of 7 months (Figure 3A). ZR-75-1 cells harbor an inactivating mutation in the tumor suppressor PTEN, which may underlie the initial dependence on AKT signaling for survival. Cells exposed to vehicle for 7 months remained sensitive to MK-2206 (Figure 3B). We analyzed mRNA expression profiles of ZR-75-1R, compared these to profiles of the parental cells, and established a gene signature of acquired AKT resistance. We then assessed the association between acquired resistance and the RAS pathway signature score by comparing RAS pathway signature score levels in the resistant derivative to parental control cells. The RAS pathway signature was significantly upregulated in ZR-75-1R cells (p = 2 × 10-7, mean fold change = 1.4) suggesting that acquired resistance to AKT inhibition was associated with increased RAS signaling.

To further assess the possibility that acquired AKTi resistance is driven by a switch from AKT signaling to Ras pathway signaling we employed reverse phase protein microarrays (RPPA) to compare phosphorylation status of AKT and ERK as surrogate markers of the activity of these pathways. Consistent with a switch from dependence on AKT signaling to dependence on signaling through RAS/ERK, the ZR-75-1R line showed decreased phosphorylation of AKT and increased phosphorylation of ERK (Figure 3C and 3D). Taken together, these data support the hypothesis that elevated RAS pathway signaling as measured by our signature is a determinant of resistance to AKT inhibition in breast cancer cell lines.

### KRAS siRNA knockdown suggests that pathway signature is more predictive of dependence on RAS dependence than KRAS mutational status

**Figure 4:**
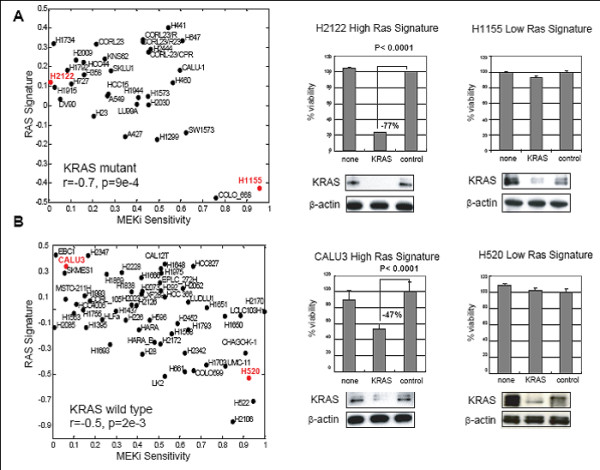
**Effect of KRAS knockdown on viability of cells harboring a KRAS mutation**. (A) KRAS mutant or (B) KRAS wild-type cells were selected for KRAS knockdown. Scatterplots indicate that these lines show variable sensitivity to MEK inhibition. The Y-axis shows RAS siganture score, and the X-axis show cell sensitivity to MEK inhibition. Lower numbers on the X-axis indicate increasing sensitivity. Cell lines in red text were treated with siRNAs targeting KRAS. Western blots were performed for KRAS and B-actin. Control = Dharmacon non-targeting siRNA pool; KRAS = siRNA targeting KRAS, none = no transfection. Bar charts show viability as measured by the ATP vialight assay. The percent viability relative to the control siRNA is shown. R = Pearson correlation coefficient, p = the corresponding P-value.

Some cell lines with a Ras mutation do not appear to have high levels of Ras signaling. A potentially important implication of this finding is that Ras mutations in cancers in some patients may not mean that these cancers are dependent on Ras signaling. To understand the relationship between the RAS pathway signature and functional dependence of cells on RAS/MEK/ERK-signaling, we next used RNA interference (RNAi) to deplete KRAS in lung cancer cell lines that exhibit high or low levels of the RAS pathway signature within both KRAS mutant and KRAS wild-type groups. The effect of KRAS knock-down on viability was assessed in H2122 (KRAS mutant, high RAS signature), H1155 (KRAS mutant, low RAS signature), Calu3 (KRAS wild-type, high RAS signature), and H520 (KRAS wild-type, low RAS signature) cell lines. These cell lines also showed differential sensitivity to PD325901 (Figure 4). Upon KRAS knockdown the growth and viability of H2122 and Calu3 were significantly decreased (77% and 47% decrease; p < 0.0001), whereas cell growth of H1155 and H520 were not markedly diminished (7% and 0% decrease; Figure 4). KRAS protein expression was reduced by at least 70% in all lines. These results indicate that not all cell lines with a KRAS mutation are dependent on RAS signaling, while some KRAS wild-type cell lines are dependent on RAS signaling. This supports the hypothesis that the RAS pathway signature is a better measure of dependence on RAS signaling compared to KRAS mutation status in lung cancer cell lines.

### Inhibition of MEK results in downregulation of the RAS pathway signature

**Figure 5:**
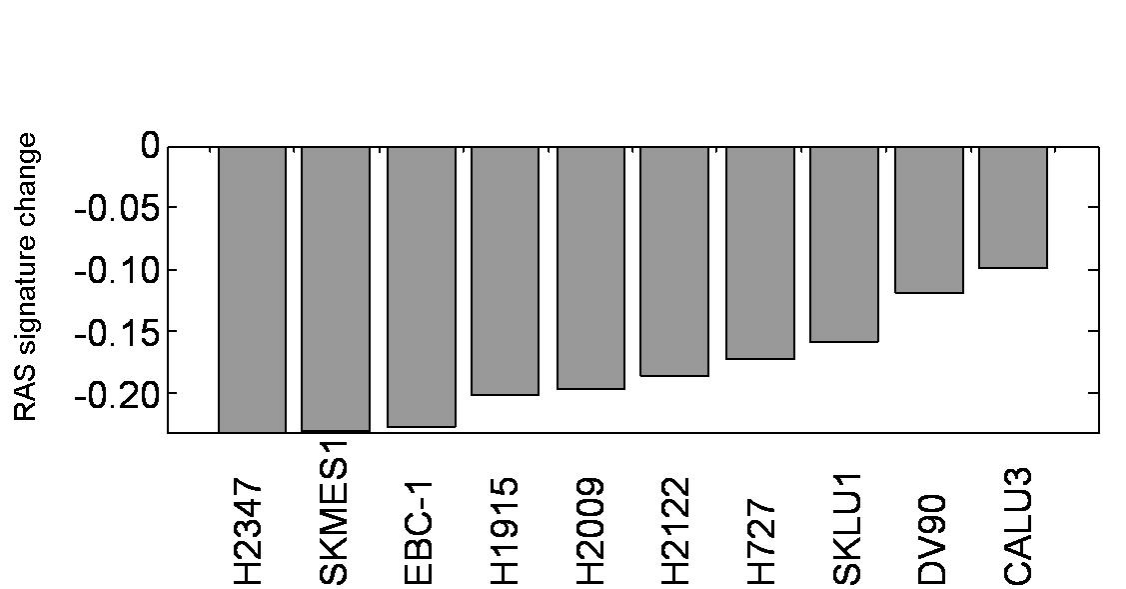
**Impact of MEK inhibition on the RAS signature across 10 lung cancer cell lines**. The Y-axis shows the RAS signature score in drug treated cell lines relative to vehicle treated controls.

The above results suggest that a core signature of RAS pathway signaling has been identified. We hypothesized that drug treatments known to inhibit key nodes of the RAS signaling pathway should downregulate the RAS pathway signature. To test this, we profiled 10 lung cancer cell lines before and after treatment with PD325901. We selected cell lines known to exhibit elevated baseline levels of the RAS pathway signature based on data shown in Figure 2A. We used a dose of 0.1 μM (known to result in efficacy in sensitive lines), and treated cells for 6 or 24 hours. As shown in Figure 5, inhibition of MEK results in significant down-regulation of the RAS pathway signature across all cell lines. These results show that the RAS pathway signature represents a gene expression module that not only reflects RAS signaling at baseline, but is responsive to RAS pathway inhibition. Therefore, the RAS pathway signature could be used as a pharmacodynamic biomarker for the assessment of pathway inhibition or activation after drug administration.

### Low RAS pathway signature score is associated with a higher Cetuximab response rate in metastatic CRC

**Figure 6:**
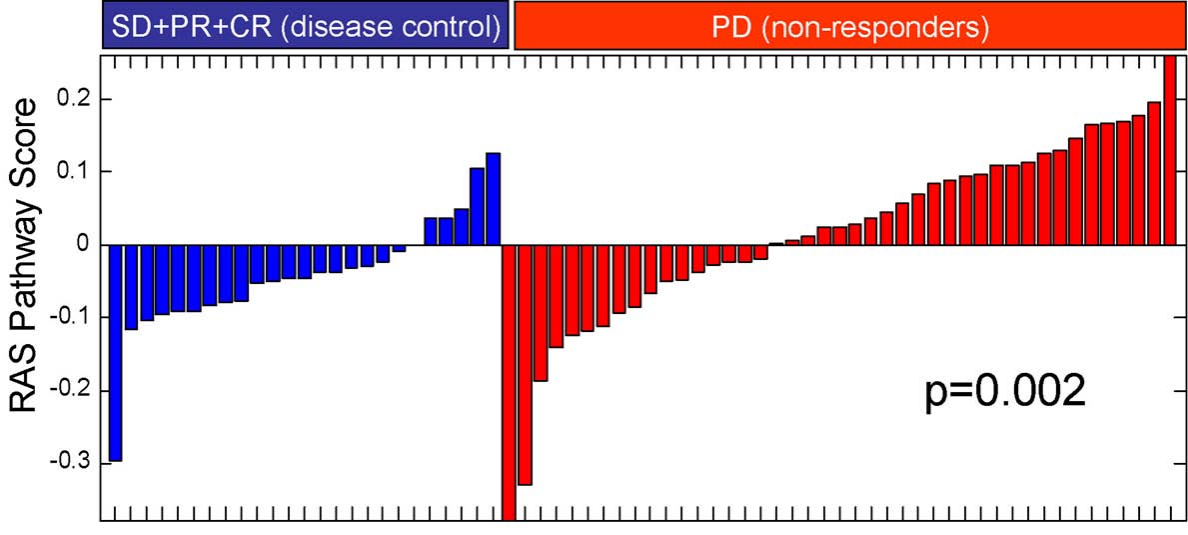
**Waterfall plot of RAS signature score in patients who experienced disease control (blue) or progressive disease (red) after Cetuximab therapy**. The Y-axis shows RAS signature score relative to the mean of all tumors in this dataset. Fisher's exact test p-value for the difference between the disease control and progressive disease groups is shown. Data is derived from [5], and includes all patients with known response.

Given the prediction of response to MEK and AKT inhibition in pre-clinical models and the translatability of the RAS pathway signature to other tumor types, we next sought to assess the predictive power of the RAS pathway signature in a clinical setting. For this purpose, we compared baseline levels of the RAS pathway signature to clinical response to the EGFR inhibitor cetuximab in a published dataset of metastatic colorectal carcinoma patients [5]. 20/25 patients (80%) experiencing disease control (stable disease, partial response, or complete response) had RAS pathway signature scores < 0, representing a significantly larger proportion compared to patients experiencing progressive disease (Figure 6, Fisher's exact test p < 0.01). This finding is consistent with the percent of patients experiencing disease control with the KRAS wild-type genotype in this dataset (89%) and with previously published findings that activated KRAS leads to cetuximab resistance in colorectal cancer [4,5,8]. As such, we conclude that the RAS pathway signature provides clinically useful information and warrants assessment as a potential predictive biomarker of response in clinical trials involving RTK/PI3K or RAS pathway inhibitors.

### Ras signature distribution and prevalence in lung and breast tumors

**Figure 7:**
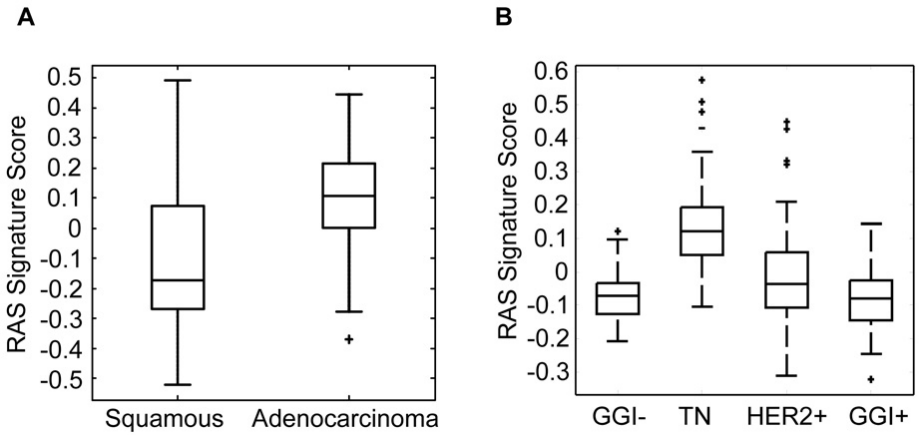
**RAS signature distribution across non-small cell lung (A) and breast (B) cancer subsets**. The Y-axis shows RAS signature score relative to the mean of all non-small cell lung (A) or breast (B) tumors in the dataset. For (B), GGI = genomic grade index [32]. GGI- = ER positive, GGI negative (surrogate for luminal A). TN = triple negative. Her2 + = high expression of Her2. GGI + = ER positive, GGI high (surrogate for luminal B).

As described above, using a RAS pathway signature score of zero as a threshold (mean value across a population), the RAS pathway signature has > 90% sensitivity for identification of KRAS mutations in lung and breast cancer and identifies additional samples with apparent RAS pathway activation in the absence of KRAS mutations. To further explore the clinical utility of the RAS pathway signature in lung and breast cancer, we assessed the distribution of and prevalence of RAS pathway signature scores across breast (Expression Project for Oncology; GSE 2109), and lung [21] tumors in publicly available datasets. We then compared this to published data regarding KRAS mutation. As shown in Figure 7A, the RAS pathway signature is significantly higher in lung adenocarcinoma compared to squamous (p < 10^-8^), with 76% of adenocarcinomas exhibiting RAS signature scores above zero compared to 30% of squamous lung tumors. This is consistent with the known association between KRAS mutations and lung adenocarcinomas, but suggests that the true proportion of lung tumors with elevated RAS pathway activity is larger than the ~15% reported prevalence of KRAS mutations in lung cancer [22,23]. In addition, the RAS pathway signature is significantly higher in ER negative breast tumors compared to other subtypes (p < 10^-10^, Figure 7B), with 79% of triple negative tumors exhibiting RAS signature scores above zero, compared to 17% of highly proliferative ER+ tumors as measured by the genomic grade index signature [24]. As breast cancers are reported to have a very low prevalence of KRAS mutations [25], these results suggest that ER negative breast tumors frequently have elevated RAS pathway activity, even in the absence of frequent KRAS mutation. Therefore, the RAS pathway signature significantly expands the estimated prevalence of lung and breast tumors with apparent RAS pathway activation, and suggests that > 75% of lung adenocarcinomas and triple negative breast cancers exhibit elevated RAS signaling.

## Conclusions

Here we describe the identification of a gene expression signature of RAS pathway activation that is coherently expressed across colorectal, lung, and breast tumors. We find that this RAS pathway signature is a high sensitivity but low specificity predictor of KRAS mutation status, as many cell line and tumor samples appear to have RAS pathway activation in the absence of mutations in KRAS. The RAS pathway signature predicts sensitivity to inhibition of MEK and resistance to inhibition of AKT in pre-clinical models, predicts resistance to Cetuximab in metastatic colorectal cancer patients, and appears to be superior to KRAS mutation status for the prediction of RAS dependence. In addition, we show that inhibition of RAS signaling through the use of a small molecule inhibitor of MEK induces a downregulation of the RAS signature. Therefore, we conclude that the RAS pathway signature is a transcriptional readout of RAS dependence that has utility for drug response prediction that is superior to KRAS mutation status.

In addition to utility as a pre-dose response predictor, inhibition of the RAS pathway signature in response to MEK inhibition indicates that the signature has utility as a pharmacodynamic/pathway inhibition readout in response to pharmacological interventions. Similarly, the RAS pathway signature could also be used to map and understand feedback regulation of RAS signaling after pharmacological inhibition of RAS or PI3K signaling components in different tumor contexts. For example, Pratilas *et al *[26] recently showed that tumors with activating mutations in Braf are insensitive to feedback downregulation of RAF signaling after MEK inhibition, and thus are dependent on MEK/ERK for survival. Future studies should include the assessment of feedback regulation of RAS and PI3K pathway components in various tumor contexts to make predictions about which inhibitor(s) should be used in which populations.

Because the RAS pathway signature was identified on the basis of coherent regulation across multiple tumor types we hypothesize that the RAS pathway signature will have utility beyond colorectal cancer, where currently the strongest clinical data exist regarding the prediction of drug response by KRAS mutations. The RAS pathway signature approximately doubles the population of "RAS active" lung tumors, and identifies triple negative breast tumors as the breast cancer subset with the highest predicted RAS pathway dependence. As such, is it likely that the RAS pathway signature will have the highest value proposition in lung and breast tumors. Because the prevalence of KRAS mutations in breast cancer is very low, yet the RAS pathway signature is coherently expressed and predicts MEK inhibitor sensitivity and AKT inhibitor resistance in breast cancer cell lines, the RAS pathway signature may have the most immediate value for clinical development in breast cancer. One clear implication of this work is that inhibitors of MEK should be clinically tested in triple negative breast tumors and NSCLC adenocarcinomas. Conversely, inhibition of PI3K pathway components like AKT without inhibition of RAS signaling is unlikely to be efficacious in triple negative breast cancers or lung adenocarcinomas, regardless of KRAS mutation status.

What could be driving elevated RAS signature scores in the absence of KRAS mutations? One possibility is that activating mutations in other canonical RAS pathway components could be responsible for elevated RAS signature in at least a subset of samples with wild-type KRAS. One candidate driver is mutant B-raf [8]; however, given the relatively high prevalence of KRAS wild-type samples with elevated RAS signature scores, it seems unlikely that this could be the sole explanation. Other candidate alterations include mutation sin H-ras, activation of receptor tyrosine kinases like EGFR, deletion of GTPase activating proteins, and other mechanisms (reviewed in [27]). Further work will be required to elucidate the key driver(s) of elevated RAS signature in various tumor contexts. If such drivers could be identified, it may be possible to design inhibitors of these drivers and design a tumor context-specific approach for targeting RAS signaling in tumors.

## Methods

### Cell culture and tumor sample sets

For cell lines used to identify the "down" arm of the signature and to assess the sensitivity of the RAS pathway signature for KRAS mutation status: cell lines were grown in ATCC recommended media in tissue culture flasks, RNA was extracted, and gene expression profiling was performed on Agilent arrays as described below.

For cell lines used to assess the relationship between the RAS pathway signature, pMEK/pERK, and MEK/AKT inhibitor response: Lung tumor-derived cell lines were obtained from various commercial vendors (ATCC, ECACC, DSMZ, HSRRB, IBL). Cells were cultured in media recommended by the vendor in tissue culture flasks. Generally, cells were passaged upon reaching 75% confluence. Sensitivity of cells to PD325901 (a derivative of CI-1040 [28], a potent and selective noncompetitive inhibitor of MEK1 and MEK2) and MK-2206, an allosteric inhibitor of AKT1, AKT2, and AKT3 [29] was determined at 8 concentrations using Cell Titer Glo (Promega; Madison, WI). Drug sensitivity values were corrected for basal growth rate of cells in order to avoid artifacts related to differential doubling times across cell lines. Given that stratification of the cell lines' relative responses is paramount, the metric should maximize the power to discriminate between individual cell line's responses. Our approach was to use a computational algorithm to find the concentration at which the population of cell lines' responses exhibited maximal variation. This was done by finding the maximum value of the variance across the concentration range tested. Cell line sensitivity was determined at this dose for each cell line in the panel and this was the primary metric of response used in for analyses. RNA was extracted from untreated cells, and gene expression profiling was performed on Affymetrix arrays as described below. Phospho-MEK and phospho-ERK levels were measured using reverse phase protein arrays as described in [20].

Downregulation of the RAS signature by MEK inhibition: Lung cancer cell lines were grown in standard media. Vehicle (DMSO) or PD325901 (0.1 μM) was added to cells for 6 or 24 hours. RNA was extracted, and gene expression profiling was performed on Affymetrix arrays as described below. Data from each post-dose group was normalized to the cell line-matched vehicle control.

For prediction of KRAS mutation status in lung tumors: 48 primary tumors were excised, frozen and embedded in OCT compound embedding medium. Tumors were macrodissected with the goal of attaining 70% tumor content before nucleic acid extraction for molecular profiling. Lung tumor mutation data were generated using mass spectrometry-based genotyping (OncoMap) [30]. RNA was extracted, and gene expression profiling was performed on Affymetrix arrays as described below.

### mRNA profiling

RNA extraction: Tissus and cell lines were homogenized within their source cryopreservation tubes using a Polytron with disposable rotostator probes. Material was homogenized in 750 to 1000 uL of 100% TRIzol. 100% Chloroform was added to the TRIzol/GITC lysate (1:5 ratio) to facilitate separation of the organic and aqueous components using the phaselock (Eppendorf) system. The aqueous supernatant was further purified using the Promega SV-96 total RNA kit, incorporating a DNase treatment during the procedure. Isolated total RNA samples were then assayed for quality (Agilent Bioanalyzer) and yield (Ribogreen) metrics prior to amplification.

Samples profiled on Affymetrix arrays: Samples were amplified and labeled using a custom automated version of the NuGEN Ovation WB protocol. Hybridization, labeling and scanning using Affymetrix ovens, fluidics stations and scanners following the protocols recommended (NuGEN). Sample amplification, labeling, and microarray processing were performed by the Rosetta Inpharmatics Gene Expression Laboratory in Seattle, WA. Samples were hybridized to the Rosetta/Merck Human RSTA Custom Affymetrix 1.0 microarray (GEO accession number **GPL6793**). Hybridization of affymetrix chips was done following the standard Affymetrix protocol. Generated .CEL files were then processed using the RMA algorithm as implemented in Affymetrix Power Tools (APT) package, using default settings and standard CDF file. Generated probeset intensities were then log10-transformed. Signature scores were calculated by averaging probesets whose gene symbols mapped to the gene sets for the "up" and "down" arms of the signature gene sets, and subtracting score for the "down" arm from the "up" arm".

Samples profiled on Agilent arrays: Samples were amplified and labeled using a custom automated version of the 5 μg RT/IVT protocol described in [31]. Sample amplification, labeling, and microarray processing were performed by the Rosetta Inpharmatics Gene Expression Laboratory in Seattle, WA. Samples were hybridized to the Rosetta/Merck Human RSTA custom Agilent 3.0 array (GEO accession number GPL3991).

### Generation of the RAS signature and assessment of signature coherence

We identified the "superset" of genes potentially sensitive to RAS deregulation by assembling the genes from the "up" arms (genes upregulated as signaling through the pathway increases) of three different publicly available RAS pathway signatures [9,12,17]. This RAS superset (consisting of 812 genes) was analyzed in publicly available cohorts of lung, colon, and breast tumors (GEO accession number GSE2109) as well as lung cancer cell lines https://array.nci.nih.gov/caarray/project/woost-00041. For each dataset, we identified a coherent gene expression module: a subset of genes whose gene expression profiles clustered together. The relationship between genes was assessed using two-dimensional clustering of the Pearson correlation matrix (using complete linkage and Euclidean distance metric as hierarchical clustering parameters). The identified gene subset is characterized by the mean/median Pearson correlation between its members of 0.40. By intersecting all four datasets, a coherent module consisting of 105 genes from the RAS superset was observed in all datasets. The genes that belonged to the module across all the datasets were selected as the "up" arm of our signature. Genes significantly anitcorrelated with the "up" arm (Pearson correlation coefficient at least -0.4) across an internal lung cancer cell line panel were selected as the "down" arm for subsequent assessment of coherence and calculation of the signature score.

The assessment of coherence is based on the opposite behavior the "up" arm, which is upregulated, and the "down" arm, which is downregulated, as signaling through the pathway increases. The purpose of coherence analysis is to show the statistical significance of the difference between the "up" and "down" arms of a signature in a new dataset. For coherence analysis, two correlation coefficients were calculated for all of the genes in both the "up" and "down" arms. First, the correlation between each gene in the "up" arm and the average of all genes in the "up" arm is calculated. Second, the anticorrelation between each gene in the "up" arm and the average of all genes in the "down" arm is calculated. This is repeated for genes in the "down" arm. If the signature is coherent, most of the genes from each arm should correlate with the corresponding arm average and anticorrelate with the average of all genes in the opposite arm. A Fisher exact test is calculated for correlation within and between arms of the signature to assess the significance of signature coherence in a new dataset. Coherence of our signature was tested in independent lung (GSE3141), colon (GSE5851), and breast (GSE2845) gene expression datasets.

### Acquired resistance to MK-2206

We generated resistant clones of a MK-2206 sensitive breast cancer cell line, ZR-75-1 (EC_50 _< 200 nM), by exposing it to increasing concentrations of MK-2206 for a period of 7 months. We obtained populations of MK-2206 resistant cells by initially treating the cells at a low concentration of MK-2206 (20 nM) and incrementally increasing the concentration as the growth rate of exposed cells reached that of cells grown in the presence of vehicle alone. Cells exhibiting robust growth in the presence of high MK-2206 (> 2 μM or 10× the original EC_50_), were grown in the absence of drug for three weeks and then confirmed as AKTi resistant in a cell proliferation assay. To control for potential non-AKT-related mechanisms of drug resistance such as up-regulation of drug efflux pumps, cells were also tested for sensitivity to taxol. Expression profiles of resistant derivatives and parental controls were generated and compared using Affymetrix arrays as described above.

### KRAS siRNA

Cells (3 × 10^5^) were seeded in 6 well plates (Costar) and transfected with KRAS and control siRNA smartpools from Dharmacon using DharmaFECT1 transfection reagent (Dharmacom #T2001-01). 18 hrs post transfection, cells were trypsinized and seeded in 96 well plates (4 × 10e3 cells/well) for the viability assay (96 hrs) or in 6 well plates for western blot analysis. Cell viability was measured using CellTiter-Glo according to manufacturer instructions (Promega; Madison, WI). For Western Blot analysis, cells were lysed 5 days post-transfection and protein levels were detected using the following antibodies: KRAS (Santa Crus #SC 30), pERK (CST #4695), pAKT(CST #4058) and β-actin (CST #4970).

## Ethics and Consent

No research on humans or animals is described in this manuscript. Lung tumor profiling data shown in Figure 1C was derived from lung tumors provided by the Moffitt Cancer Center and all patients were appropriately consented.

## Competing interests

At the time of authoring this manuscript, all authors were employees of Merck & Co.

## Authors' contributions

AL, MN, MC, TZ, HD and JW performed data analysis, interpreted results, and created figures for the manuscript. RK and JF performed acquired resistance studies. BA and BR led cell line drug response experiments. BH and MC led experiments to assess RAS signature inhibition after MEK inhibitor treatment. JA and KN performed Kras siRNA studies. CP and BL performed reverse phase protein array experiments. PH provided scientific direction, critical review, and data interpretation. JW led authoring of the manuscript. All authors read and approved the final manuscript.

## Pre-publication history

The pre-publication history for this paper can be accessed here:

http://www.biomedcentral.com/1755-8794/3/26/prepub

## Supplementary Material

Additional file 1 **Coherence of RAS pathway signatures**. Correlation plots showing the relationship between the "UP" and "DOWN" arms of various RAS pathway signatures. A. Bild et al [9], B. Sweet-Cordero et al [12], C. Blum et al [17], D. The ras pathway signature described in this publication. The Y-axis shows the mean log(10) ratio of the genes within the "DOWN" arm of a signature, and the X-axis shows the mean log(10) ratio of the genes within the "UP" arm of a signature. Each dot represents a tumor in the ExpO lung dataset. If a signature is coherent, a pattern of anti-correlation is expected. Click here for file

Additional file 2 **Gene overlap in publicly available datasets**. Venn diagram showing the overlap in genes within the 812 gene RAS "superset" that are correlated with one another in publicly available datasets. Datasets are listed, number of samples is shown in parentheses. Click here for file

Additional file 3 **RAS signature genes**. Genes comprising the RAS signature. Click here for file

Additional file 4 **RAS signature coherence**. p-value by Fisher exact test for signature coherence Click here for file
