# Supplementary figures and images for: A gene expression signature of RAS pathway dependence predicts response to PI3K and RAS pathway inhibitors and expands the population of RAS pathway activated tumors
Source: BMC Med Genomics. 2010 Jun 30;3:26. doi: 10.1186/1755-8794-3-26 (PMC2911390; doi:10.1186/1755-8794-3-26)

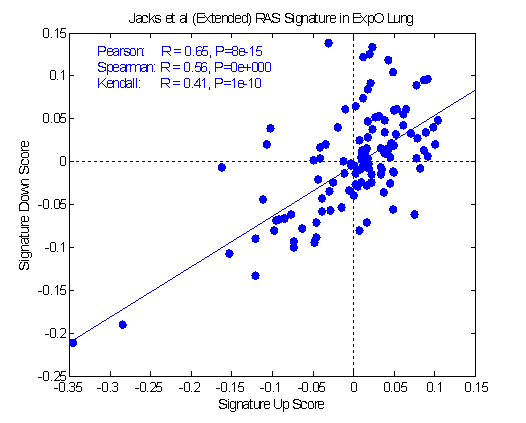

Supplement: Additional file 1 — Coherence of RAS pathway signatures. Correlation plots showing the relationship between the "UP" and "DOWN" arms of various RAS pathway signatures. A. Bild et al [9], B. Sweet-Cordero et al [12], C. Blum et al [17], D. The ras pathway signature described in this publication. The Y-axis shows the mean log(10) ratio of the genes within the "DOWN" arm of a signature, and the X-axis shows the mean log(10) ratio of the genes within the "UP" arm of a signature. Each dot represents a tumor in the ExpO lung dataset. If a signature is coherent, a pattern of anti-correlation is expected. [file 1755-8794-3-26-S1.TIFF]

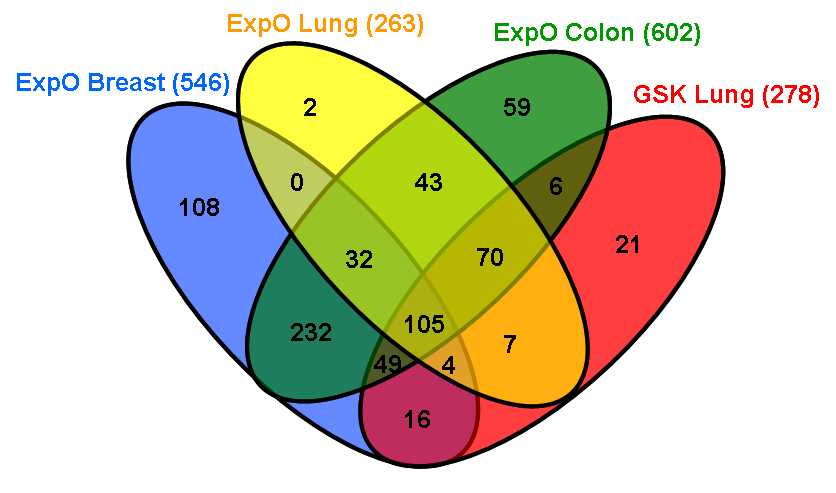

Supplement: Additional file 2 — Gene overlap in publicly available datasets. Venn diagram showing the overlap in genes within the 812 gene RAS "superset" that are correlated with one another in publicly available datasets. Datasets are listed, number of samples is shown in parentheses. [file 1755-8794-3-26-S2.TIFF]
